# Developmental Dopamine Loss Rewires Striatal Circuits to Promote Locomotion

**DOI:** 10.21203/rs.3.rs-7401124/v1

**Published:** 2025-09-15

**Authors:** Jie Dong, Breanna T. Sullivan, Victor M. Martinez Smith, Lupeng Wang, Lulu Tian, Justin Kung, Bin Song, Shirong Lin, Andreanna Le, Lixin Sun, Lisa Chang, Jinhui Ding, Weidong Le, Jun Jia, Huaibin Cai

**Affiliations:** National Institutes of Health; National Institutes of Health; National Institutes of Health; National Institutes of Health; National Institutes of Health; National Institutes of Health; National Institutes of Health; National Institutes of Health; National Institutes of Health; National Institutes of Health; National Institutes of Health; National Institutes of Health; Zhejiang University School of Medicine; National Institutes of Health; National Institutes of Health

**Keywords:** ALDH1A1, Kremen1, dopaminergic neurons, Parkinson’s disease, Pitx3, striatal projection neurons, direct-pathway, indirect-pathway, optogenetics, neuromodulation

## Abstract

**Background:**

Motor symptoms of Parkinson’s disease (PD) primarily result from the degeneration of nigrostriatal dopaminergic neurons (DANs), particularly the Aldehyde Dehydrogenase 1A1-positive (ALDH1A1^+^) subpopulation. *Pitx3*-deficient mice exhibit selective developmental loss of ALDH1A1^+^ DANs but paradoxically display hyperlocomotion, suggesting compensatory changes in striatal circuitry. The dorsal striatum contains four main types of spiny projection neurons (SPNs): patch (or striosome) and matrix subtypes of both direct-pathway (dSPNs) and indirect-pathway (iSPNs). Activation of patch dSPNs suppresses locomotion by inhibiting ALDH1A1^+^ DANs.

**Methods:**

We combined RNAscope *in situ* hybridization with SPN subtype-specific reporter mice to quantify patch and total dSPNs and iSPNs in *Pitx3*-deficient and control mice. Three patch SPN reporter lines (*Kremen1*^2A-Cre^, *Nr4a1*-*GFP*, and *Pdyn*^IRES-Cre^) were used to map projections. Optogenetic stimulation was performed in freely moving mice to assess the behavioral effects of activating patch dSPNs and iSPNs.

**Results:**

*Pitx3-*deficient mice showed no change in the overall dSPN:iSPN ratio but exhibited a marked shift in the patch dSPN:patchy iSPN ratio, which decreased from 1.7 in control mice to 0.7 in the *Pitx3-*deficient group. Accordingly, patch dSPN projections to the *substantia nigra pars reticulata* (SNr) were reduced, whereas patch iSPN projections to the *globus pallidus externus* (GPe) were enhanced. Notably, while optogenetic stimulation of patch dSPNs and iSPNs suppressed locomotion in control mice, the same stimulation promoted locomotion in *Pitx3*-deficient mice.

**Conclusions:**

Our findings reveal a selective reorganization of patch SPNs in response to developmental loss of ALDH1A1^+^ DANs, characterized by reduced patch dSPN and enhanced patch iSPN influence. This shift may underlie the paradoxical hyperlocomotion observed in *Pitx3*-deficient mice and provides insight into circuit-level adaptations with potential therapeutic relevance for PD.

## Background

Patients with Parkinson’s disease (PD) experience progressive motor symptoms, including resting tremor, slowed movement, and impaired posture and balance [1]. In addition to these motor issues, they often suffer from non-motor symptoms such as depression and dementia [2]. While medications and surgical interventions can improve motor function, long-term medication can lead to severe side effects, including dyskinesia and impulse control disorders [3, 4]. Therefore, there is a continuing need for new mechanistic insights and therapeutic strategies to improve treatment options for the growing number of PD patients [5].

The motor symptoms of PD are primarily associated with the degeneration of midbrain dopaminergic neurons (DANs) in the *substantia nigra compacta* (SNc) [6, 7], particularly the Aldehyde Dehydrogenase 1A1-positive (ALDH1A1^+^) subset in its ventral tier [8–11]. The ALDH1A1^+^ SNc DANs accounts for 60–70% DANs in human and rodent SNc and mainly project to the dorsal portion of dorsal striatum [8, 10, 12, 13]. In naturally occurring Pituitary homeobox 3 (*Pitx3*)-deficient aphakia (or *Pitx3*^ak/ak^) mice, midbrain DAN differentiation remains unaffected at embryonic day 11.5 (E11.5) [14]. However, a noticeable reduction in tyrosine hydroxylase (TH)-positive cells destined for the SNc emerges by E12.5 [14], leading to a significant loss of SNc DANs by postnatal day 3 [15]. In contrast, DANs in the ventral tegmental area (VTA) remain largely unaffected [14–17]. Notably, the depleted SNc DANs in these mice predominantly belong to the ALDH1A1^+^ DAN subpopulation [18]. Interestingly, while acute genetic ablation of ALDH1A1^+^ DANs in adult mice slows locomotion [12], the developmental loss of these neurons in *Pitx3*^ak/ak^ mice does not decrease nighttime movement [19]. Instead, it increases daytime activity [19]. This adaptation likely involves striatal projection neurons (SPNs) in the dorsal striatum, which are the primary targets of midbrain DAN input [20, 21]. Investigating these compensatory mechanisms could provide valuable insights into neuromodulatory strategies for addressing PD-related motor deficits.

In the dorsal striatum, SPNs are broadly classified into two major subtypes based on their molecular identity and projection targets: direct-pathway SPNs (dSPNs), which express dopamine receptor D1 (DRD1) and project to the internal globus pallidus (GPi) and substantia nigra pars reticulata (SNr), and indirect-pathway SPNs (iSPNs), which express dopamine receptor D2 (DRD2) and project to the external globus pallidus (GPe) [22–25]. Both dSPNs and iSPNs are distributed across two complementary compartments—the patch (or striosome) and the matrix, which differ in anatomical organization, gene expression profiles, and connectivity patterns [26–30]. Notably, SPNs with molecular characteristics of patch neurons can also be found scattered within the matrix compartment; these are referred to as “exo-patch” SPNs [31]. Since molecularly labeling does not distinguish between canonical patch and exo-patch locations, we use the term “patchy SPNs” to collectively refer to both populations in this study. Among midbrain DANs, ALDH1A1^+^ DANs in the SNc receive the strongest monosynaptic inhibitory input from patchy dSPNs [12, 32]. Recent studies have shown that activation of patchy dSPNs suppresses locomotion via inhibition of ALDH1A1^+^ DANs and reduction of dopamine release [33–35], whereas patchy iSPNs appear to facilitate movement, highlighting compartment- and cell type-specific functional roles [33, 35]. However, it remains unclear how the developmental loss of ALDH1A1^+^ DANs in *Pitx3*^ak/ak^ mice affects the organization and function of SPNs, particularly patchy SPNs.

In this study, we examine the structural and functional reorganization of SPNs, with a focus on patchy SPNs, in *Pitx3*^ak/ak^ mice. We analyze the composition and distribution of molecularly defined patchy dSPNs and iSPNs in the dorsal striatum and assess their projection patterns to the GPe and SNr. Additionally, we use optogenetic approaches to selectively activate patchy dSPNs and iSPNs to determine their impact on locomotion.

## Methods

### Animals

All mouse procedures were approved by the Institutional Animal Care and Use Committee (IACUC) of the Intramural Research Program at the National Institute on Aging (NIA), NIH, and conducted in accordance with institutional and NIH guidelines. All mouse lines were maintained as heterozygotes on a C57BL/6J background. The following strains were obtained from The Jackson Laboratory: *Pitx3*^ak/ak^ (Stock No: 000942) [17], *Drd1–*tdTomato (Stock No: 016204) [36], *Pdyn*^IRES–Cre^ (Stock No: 027958) [37], and Ai14 (Stock No: 007908) [38]. The *Pitx3*^ak/ak^ mice were backcrossed with C57BL/6J for more than five generations. The *Kremen1*^2A – Cre^ knock-in mice [33] were generated by Shanghai Model Organisms Inc. (Shanghai, China), while the *Nr4a1–*eGFP (Stock No: 036737-UCD) transgenic mice [39] were obtained from the Mutant Mouse Resource & Research Centers (MMRRC).

To examine the projections of patchy dSPNs and iSPNs, three patchy SPN mouse lines (*Kremen1*^2A – Cre^, *Pdyn*^IRES–Cre^, and *Nr4a1–*eGFP) were bred into the *Pitx3*^ak/ak^ background, and their littermates *Pitx3*^+/ak^ were used as control in the experiments. Both male and female mice were used in all experiments. Mice were group-housed (2–5 per cage) under a 12-hour light/dark cycle with ad libitum access to water and standard chow. Behavioral experiments were conducted during the light phase. Littermates were randomly assigned to experimental groups before study onset.

#### RNA In Situ Hybridization and Image Analysis

RNA *in situ* hybridization (RNAscope, ACDBio) was performed to detect *Drd1*, *Drd2*, and *Kremen1* mRNAs in the dorsal striatum of adult C57BL/6J mice. Mice were euthanized with CO_2_ inhalation. Brains were fresh-frozen on dry ice and stored at − 80°C. Coronal sections (12 μm) were cut on a cryostat (Leica Biosystems) and stored at − 80°C.

RNAscope was performed using the Multiplex Fluorescent Reagent Kit v2 per the manufacturer’s instruction. Probes used included: *Drd1* (Cat. No. #401901), *Drd2* (Cat. No. #406501), and *Kremen1* (Cat. No. #425771). Images were acquired on a Zeiss LSM 780 laser scanning confocal microscope with 20× or 40× objectives. Five sections were analyzed spanning a rostro-caudal range from approximately 1.34 mm to − 0.34 mm relative to Bregma.

Image analysis was conducted using Imaris v10.0 (Bitplane, Belfast, UK). Dorsal and ventral striatal regions were delineated using the Allen Brain Atlas. Spatial patches were defined as clusters of at least five *Kremen1*^+^ SPNs with a minimum density of 200 cells/mm^2^. Channels (*Drd1*, *Drd2*, *Kremen1*, and DAPI) were analyzed with consistent parameters within each batch; minor adjustments across batches ensured optimal quantification. DAPI^+^ cells were classified as *Drd1*^+^ (dSPNs) or *Drd2*^+^ (iSPNs) based on mean fluorescence intensity. Overlap with *Kremen1* signal was used to quantify patchy dSPNs and iSPNs. Data points represent averages across multiple bilateral striatal sections.

### Immunohistochemistry

Mice were anesthetized with pentobarbital and transcardially perfused with PBS followed by 4% paraformaldehyde (PFA). Brains were post-fixed overnight at 4°C, then cryoprotected in 30% sucrose (PBS-buffered) for ≥ 48 hours. Coronal sections (40 μm) were cut and stored in PBS with 4% sodium azide at 4°C.

Sections were blocked for 1 hour at room temperature in 10% normal donkey serum, 0.5% BSA, and 0.3% Triton-X-100. They were incubated with primary antibodies overnight or for 48 hours at 4°C, washed (3 × 10 min, PBS), and then incubated with secondary antibodies for 1 hour. Some sections were counterstained with DAPI (0.5 mg/mL, 1 min, Invitrogen, D1306) and mounted using ProLong Gold Antifade Mountant (Life Technologies). Images were captured using a Zeiss LSM 780 or LSM 980 confocal microscope.

Primary antibodies used: polyclonal rabbit anti-PITX3 (1:500) [40], rabbit anti-TH (Pel-Freez Biologicals, P40101; 1:1000), goat anti-ALDH1A1 (R&D system; 1:1000), guinea pig anti-PV (Swant, GP72; dilution 1:1000), goat anti-ChAT (AB144P, Millipore; dilution 1:500) and rat anti-CTIP2 (abcam, ab18465, 1:500). Secondary antibodies (Life Technologies) were selected for appropriate fluorophore spectra.

### Quantification of TH and ALDH1A1 Neurons

Midbrain sections (40 μm) spanning Bregma – 2.70 mm to – 3.80 mm (seven sections per mouse) were stained for TH, ALDH1A1 and DAPI. Z-stack confocal images were captured at 20× magnification. Neurons in the SNc and VTA were quantified using the “Detect Cells” functions in NeuroInfo (MBF Bioscience). Regions were manually delineated using previously established anatomical landmarks. Total neuron numbers were estimated using “Area Under the Curve” analysis in GraphPad Prism 10.

#### Quantification of TH^+^ and ALDH1A1^+^ Axon Terminal in the Striatum

Five to six striatal sections across a rostro-caudal series (approximately 1.42mm to −0.2 mm relative to Bregma) were co-stained for TH and ALDH1A1. Images were acquired with 10 × objective on the Zeiss LSM780. ALDH1A1^+^ areas in the dorsal striatum were outlined using intensity threshold in ImageJ. For each section, the percentage of ALDH1A1^+^ area relative to total striatal area was calculated.

### Quantification of Nr4a1-eGFP cells in the dorsal striatum

In *Nr4a1*-eGFP; *Drd1*-tdTomato mice on either *Pitx3*^+/ak^ or *Pitx3*^ak/ak^ backgrounds, striatal sections spanning from Bregma 1.34mm to −0.34mm (5 sections per each mouse) were stained for CTIP2 and imaged using a Zeiss LSM 780 laser scanning confocal microscope with a 10× objective. Dorsal striatal regions were delineated according to the Allen Brain Atlas. Cell quantification was performed using Imaris v10.0 (Bitplane, Belfast, UK). CTIP2^+^ cells were identified as SPNs and further classified as tdT^+^ (dSPNs) and tdT-negative (iSPNs) based on median fiuorescence intensity. Overlap with GFP^+^ signal was used to quantify Nr4a1-eGFP^+^ dSPNs and iSPNs. Minor threshold adjustments were applied across batches to ensure optimal quantification.

### Quantification of fluorescence intensity and cell density

Fluorescence intensity and cell density were quantified using ImageJ. For each mouse, 3–8 striatal or midbrain sections at different Bregma levels were analyzed. For SNr and GPe regions, mean RFP or GFP fiuorescence intensity was calculated by delineating the regions of interest based on anatomical landmarks in the Allen Brain Atlas.

To assess fluorescence distribution, “Plot Profile” function was used to generate intensity plots along the drawn line. Colocalization analysis between GFP^+^ and PV^+^ signals was performed using the “Coloc2” function in ImageJ to calculate Manders’ coefficients M1 and M2, indicating the proportion of GFP signal overlapping with PV and vice versa.

For cell density analysis, PV^+^ and ChAT^+^ cells were automatically counted in the dorsal striatum by setting the particle diameter. The counting area was measured, and cell density was calculated as the number of positive cells per mm^2^.

### Stereotaxic Viral Injection and Optic Fiber Implantation

Stereotaxic surgeries were performed under aseptic conditions. Mice (2–4 months old) were anesthetized with 1–2% isoflurane and placed in a stereotaxic frame (Kopf Instruments). A total of 700 nL of AAV was bilaterally injected into the dorsal striatum (AP: +0.9 mm; ML: ±2.2 mm; DV: −2.5 mm) at 75 nL/min using a microinjector (Stoelting). Vectors included AAV1-EF1a-double fioxed-hChR2(H134R)-EYFP (#20298) and AAV1-Ef1a-DIO-EYFP (#27056) from Addgene (Watertown, MA, USA). The needle was left in place for 5 minutes post-injection before withdrawn. Incisions were closed and mice recovered in home cages.

After three weeks, optical fibers (200 μm core, 0.39 NA; Thorlabs) were bilaterally implanted targeting either the dorsal striatum (AP: +1.0 mm; ML: ±1.5 mm; DV: −2.2 mm to – 2.7 mm) or SNr (AP: −3.1 mm; ML: ±1.5 mm; DV: −4.1 mm DV). Fibers were secured with radiopaque adhesive cement (C&B METABOND, Parkell) and incisions were sealed with Vetbond tissue adhesive (3M). Mice recovered for ≥ 1 week before testing.

### Open-Field Spontaneous Locomotion

Freely moving mice were assessed for spontaneous locomotion via video tracking. Prior to testing, mice were habituated for 30 minutes. The arena (50 × 50 cm, opaque gray) was lit diffusely using an enclosed 20W lamp. Mice were recorded for 30 minutes at 30 Hz from above using a digital camera. For analysis, the arena was divided into a center zone (central 25 × 25 cm) and a surrounding zone. Data on velocity, distance, and time traveled were analyzed with EthoVision XT (Noldus). For beam break tests, mice were placed in a 43 × 23 cm arena with infrared sensors. Chambers were cleaned with 50% ethanol between trials.

### Optogenetics in the Open Field Test

Light delivery was controlled using an LED source and commutator (PlexBright, Plexon) connected via a patch cable (200 μm, 0.39 NA). Connections were made with ceramic sleeves (Thorlabs). Light power (465 nm) was calibrated to 3 mW at the fiber tip (Thorlabs PM100D). For ChR2 activation, 5-ms light pulses were delivered at varying frequencies and durations controlled via a TTL signal generator (OPTG-4, Doric Lenses).

Mice were habituated for 30 minutes before testing. The arena (50 × 50 cm, transparent walls) was cleaned between sessions. Mice were recorded from both top and side views (Logitech cameras) at 15 Hz. After a 3-minute baseline, optogenetic stimulation was applied bilaterally in 10 sec ON / 1 min OFF cycles. Video and TTL signals were synchronized using Synapse software (TDT). Locomotion metrics were analyzed in EthoVision XT.

### Statistical Analysis

Statistical analyses were performed in GraphPad Prism 10 and custom MATLAB scripts (MathWorks). Data are presented as mean ± SEM. Specific statistical tests are reported in figure legends. Significance was assessed using two-tailed t-tests and one-way ANOVA, with thresholds of *p* < 0.05 (*), *p* < 0.01 (**), *p* < 0.001 (***), and *p* < 0.0001 (****).

## Results

### Pitx3^ak/ak^ mice exhibit hyperlocomotion despite the loss of ALDH1A1^+^ DANs

**Figure 1:**
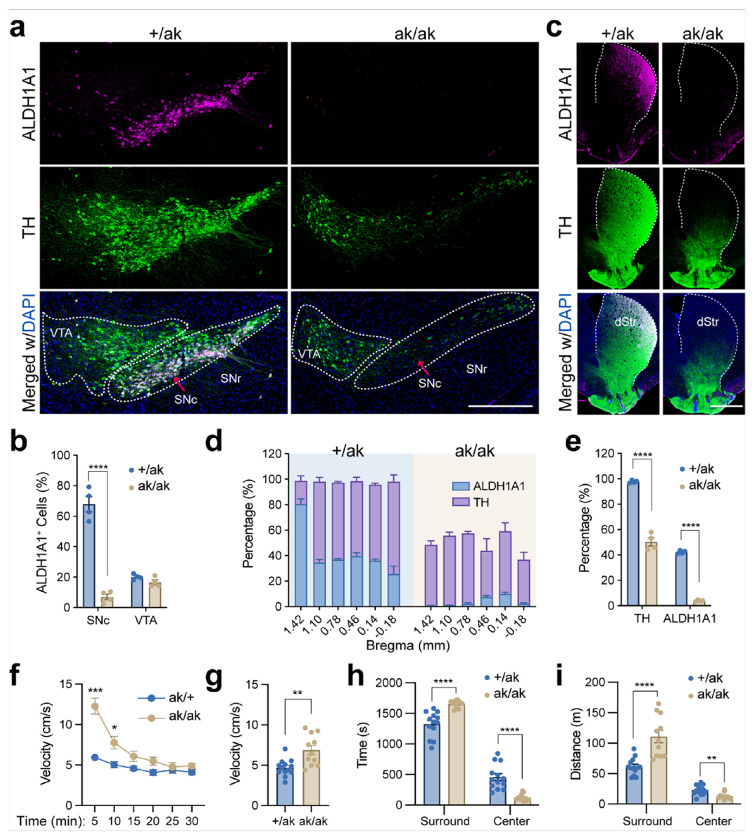
Preferential loss of ALDH1A1^+^ SNc DANs and hyperlocomotion in *Pitx3*^ak/ak^ mice. **a** Representative image showing ALDH1A1 (magenta), TH (green), and DAPI (blue) immunolabeling in the midbrain of *Pitx3*^+/ak^ and *Pitx3*^ak/ak^ mice. Dashed lines outline the *substantia nigra pars compacta* (SNc) and ventral tegmental area (VTA). Scale bar, 200 μm. **b** Percentage of ALDH1A1^+^ cells among TH^+^ cells in the SNc and VTA. Unpaired two-tailed t test, SNc: t(6) = 11.23, ****p < 0.0001; VTA: t(6) = 1.989, p = 0.094; n = 4 mice per group. **c** Representative images of ALDH1A1 (magenta), TH (green), and DAPI (blue) staining in the dorsal striatum. Dashed lines indicate the boundaries of the dorsal striatum. Scale bar, 1 mm. **d** Quantification of ALDH1A1^+^ and TH^+^ signal as a percentage of total striatal area across multiple bregma levels (n = 4 mice per genotype). **e** Group averages of the percentage of ALDH1A1^+^ and TH^+^ areas relative to the total striatum area from (**d**). TH: +/ak, 97.76 ± 0.53%; ak/ak, 50.34 ± 3.21%; unpaired two-tailed t-test, t(6) = 14.57, ****p < 0.0001. ALDH1A1: +/ak, 42.20 ± 0.61%; ak/ak, 4.00 ± 0.27%; t(6) = 57.21, ****p < 0.0001. **f** Locomotor velocity over time during a 30-min open-field test. Two-way ANOVA: time effect, F(2.42, 53.27) = 75.72, ****p < 0.0001; genotype effect, F(1, 22) = 13.02, **p = 0.0016; interaction, F(5, 110) = 28.59, ****p < 0.0001. Multiple comparisons between genotypes: 5 min, ***p = 0.0003; 10 min, *p = 0.042. **g** Average velocity across the 30-min session from (f). Unpaired two-tailed t-test, t(22) = 3.608, **p = 0.0016. **h** Time spent in surround and center zones of the open field. Unpaired t test, t(22) = 5.174, ****p < 0.0001. **i** Distance traveled in the surround and center zones. Surround: t(22) = 4.817, ****p < 0.0001; center: t(22) = 3.639, **p = 0.0014. Sample sizes: *Pitx3*^+/ak^, n = 7 males, 6 females; *Pitx3*^ak/ak^, n = 4 males, 7 females (**f–i**). Data are presented as mean ± SEM. ****p < 0.0001; ***p < 0.001; **p < 0.01; *p < 0.05.

Consistent with previous findings [14, 16, 17], PITX3 expression was completely absent in midbrain DANs of *Pitx3*^ak/ak^ mice (**Supplementary Fig. S1**). Loss of PITX3 resulted in a selective depletion of ALDH1A1^+^ DANs in the ventral SNc, whereas ALDH1A1– SNc DANs and VTA DANs remained largely intact (Fig. 1a, 1b). Accordingly, ALDH1A1^+^ axon projections were severely reduced in the dorsal striatum of *Pitx3*^ak/ak^ mice (Fig. 1c–e).

Unexpectedly, *Pitx3*^ak/ak^ mice displayed hyperactivity in the open-field test compared to littermate controls (Fig. 1f, 1g). Mutant mice also spent significantly more time and traveled longer distances in the peripheral zone relative to the center (Fig. 1h, 1i), indicative of elevated anxiety-like behavior. This paradoxical increase in locomotor activity despite the loss of ALDH1A1^+^ DANs suggests a possible developmental compensatory mechanism, potentially involving circuit-level reorganization of SPNs.

### Significant reduction in patchy dSPNs and increase in patchy iSPNs in Pitx3^ak/ak^ mice

**Figure 2:**
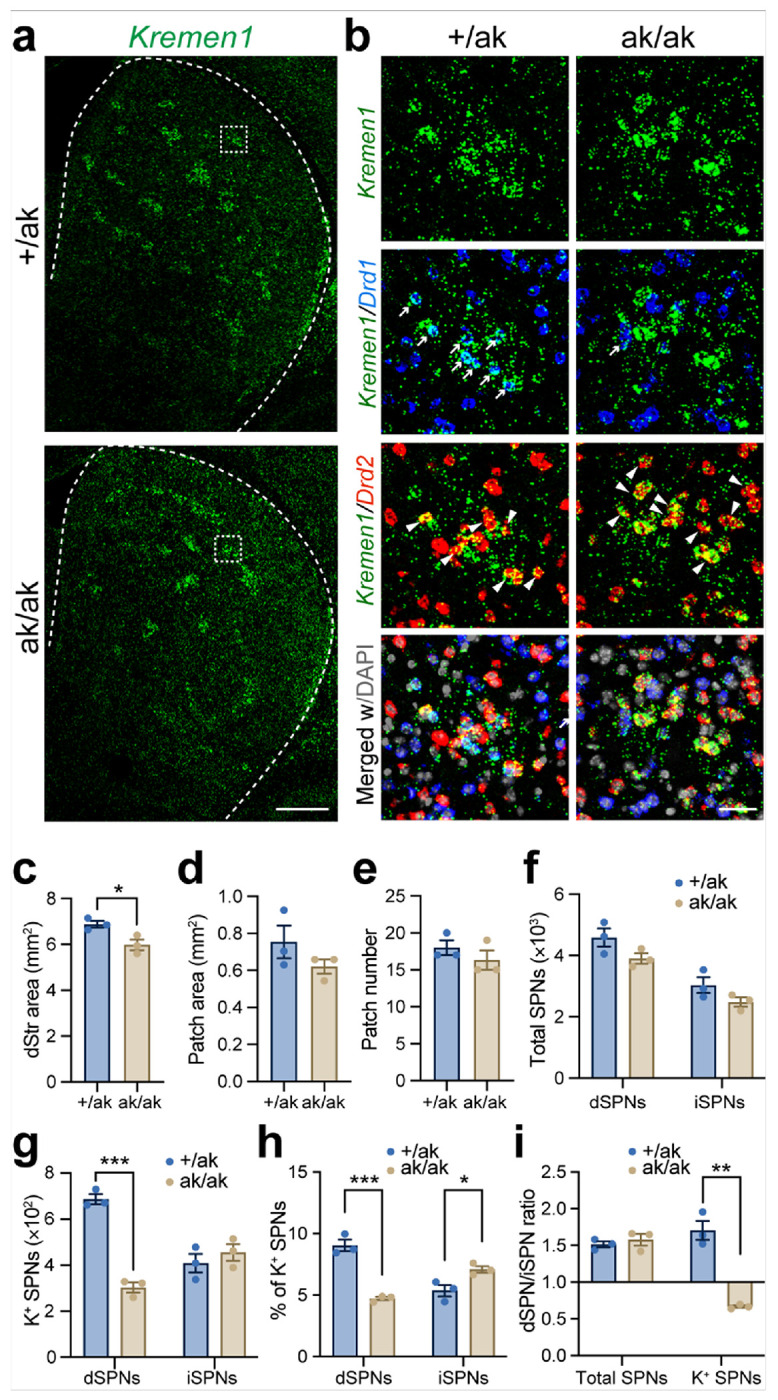
Altered numbers of *Kremen1*^+^ dSPNs and iSPNs in *Pitx3*^ak/ak^ mice **a, b** Representative confocal images showing RNAscope labeling of *Kremen1*(green), *Drd1* (blue), *Drd2* (red), and DAPI (gray) in the dorsal striatum (dStr) of *Pitx3*^+/ak^ and *Pitx3*^ak/ak^ mice. Right panels show high-magnification views of boxed regions from the left panels. Scale bars: 500 μm (left), 50 μm (right). **c** Quantification of total dStr area. Unpaired two-tailed t-test: t(4) = 3.239, *p = 0.032. **d, e** Quantification of spatial patch area (d) and patch number (e) in *Pitx3*^+/ak^ and *Pitx3*^ak/ak^ mice. Unpaired t-test, patch area: t(4) = 1.379, p = 0.24; patch number: t(4) = 1.000, p = 0.37. Spatial patches were defined as clusters of ≥ 5 Kremen1^+^ SPNs with local density ≥ 200 cells/mm^2^. **f** Total numbers of dSPNs and iSPNs in the dStr. Unpaired t test, dSPNs: t(4) = 1.993, p = 0.12; iSPNs: t(4) = 1.845, p = 0.14. **g** Numbers of *Kremen1*^+^ dSPNs and iSPNs in the dStr. *Kremen1*^+^ dSPNs: t(4) = 12.56, **p = 0.0002; *Kremen1*^+^ iSPNs: t(4) = 0.87, p = 0.43. **h** Proportion of *Kremen1*^+^ dSPNs among total dSPNs, and *Kremen1*^+^ iSPNs among total iSPNs. dSPN ratio: t(4) = 8.835, ***p = 0.0009; iSPN ratio: t(4) = 3.187, *p = 0.033. **i** Ratio of dSPNs to iSPNs and of *Kremen1*^+^ dSPNs to *Kremen1*^+^ iSPNs. dSPN/iSPN ratio: t(4) = 0.68, p = 0.54; *Kremen1*^+^ dSPN/iSPN ratio: t(4) = 7.992, **p = 0.0013. Data are presented as mean ± SEM. n = 3–5 mice per group. ***p < 0.001; **p < 0.01; *p < 0.05.

To assess changes in SPN subtypes, we performed multiplexed RNAscope *in situ* hybridization using probes for *Drd1*, *Drd2*, and *Kremen1*, which label dSPNs, iSPNs, and a subset of patchy SPNs, respectively [33, 41]. This approach allowed us to map the composition and distribution of SPN subtypes in the dorsal striatum of *Pitx3*^ak/ak^ and littermate control mice (Fig. 2a, 2b). Although *Pitx3*-deficiency led to a significant reduction in the overall surface area of the dorsal striatum (Fig. 2c), it did not alter the size or number of patch-like structures (Fig. 2d, 2e), nor did it affect the total numbers of dSPNs or iSPNs (Fig. 2f).

Notably, the number of *Kremen1*^+^ patchy dSPNs was substantially reduced in *Pitx3*^ak/ak^ mice, whereas the number of patchy iSPNs showed a slight increase (Fig. 2g). Quantification of the proportion of *Kremen1*^+^ cells within the total dSPN and iSPN populations revealed opposite trends: the fraction of patchy dSPNs was significantly decreased, while that of patchy iSPNs was increased (Fig. 2h). As a result, the ratios of patchy dSPNs to patchy iSPNs was reversed, shifting from 1.7 in control mice to 0.7 in *Pitx3*^ak/ak^ mice (Fig. 2i).

**Figure 3:**
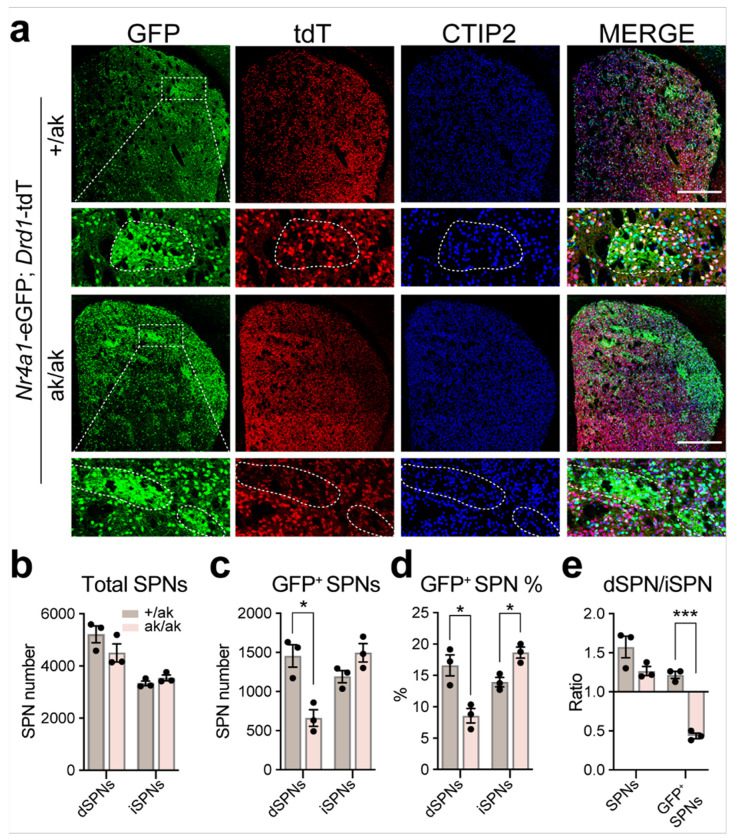
Altered numbers of *Nr4a1*-eGFP^+^ dSPNs and iSPNs in *Pitx3*^ak/ak^ mice **a** Representative coronal sections of the dorsal striatum from *Nr4a1*-eGFP; *Drd1*-tdT mice on *Pitx3*^+/ak^ and *Pitx3*^ak/ak^ backgrounds, stained for GFP (green), tdT (red) and CTIP2 (blue). Row 2 and 4 show magnified views of the dotted rectangles in row 1 and 3, respectively. Patch regions are outlined in row 2 and 4. Scale bars: 500 μm **b** Quantification of average numbers of dSPNs and iSPNs in the hemi-dorsal striatum of *Pitx3*^+/ak^ and *Pitx3*^ak/ak^ mice. Unpaired t test, n = 3 mice per each group; dSPNs: t(4) = 1.514, p = 0.2046; iSPNs: t(4) = 1.434, p = 0.2249. **c** Quantification of average numbers of *Nr4a1*-eGFP^+^ dSPNs and iSPNs in the hemi-dorsal striatum. Unpaired t test, n = 3 mice per each group; dSPNs: t(4) = 4.450, *p = 0.0112; iSPNs: t(4) = 2.185, p = 0.0942. **d** Percentage of *Nr4a1*-eGFP^+^ SPNs among total SPNs in the dorsal striatum. Unpaired t test, n = 3 mice per each group; dSPNs: t(4) = 3.978, *p = 0.0164; iSPNs: t(4) = 4.063, *p = 0.0153. **e** Ratio of dSPNs to iSPNs and *Nr4a1*-eGFP^+^ dSPNs to *Nr4a1*-eGFP^+^ iSPNs in the dorsal striatum. Unpaired t test, n = 3 mice per each group; dSPNs/iSPNs: t(4) =2.07, p = 0.1073; *Nr4a1*-eGFP^+^ dSPNs/*Nr4a1*-eGFP^+^ iSPNs: t(4) =14.03, ***p = 0.0001. Data are presented as mean ± SEM. n = 3 mice per group. ***p < 0.001; *p < 0.05.

These findings were corroborated in an independent patchy SPN reporter model, *Nr4a1*-eGFP; *Drd1*-tdTomato; *Pitx3*^ak/ak^ mice, in which patchy SPNs were labeled by GFP [39], dSPNs by tdTomato (tdT) [36], and iSPNs identified as CTIP2-positive but tdT-negative (CTIP2^+^ /tdT^−^) cells (Fig. 3a–e). CTIP2 is a general marker for SPNs [42]. By contrast, we observed no apparent changes in PV^+^ and ChAT^+^ interneurons in the dorsal striatum of *Pitx3*^ak/ak^ mice (**Supplementary Fig. S2**).

Together, these findings from two independent models demonstrate that PITX3 loss selectively alters patchy SPN composition, characterized by a decrease in patchy dSPNs and a relative increase in patchy iSPNs.

### Reduced SNr projections from patchy dSPNs and increased GPe projections from patchy iSPNs in Pitx3^ak/ak^ mice

**Figure 4:**
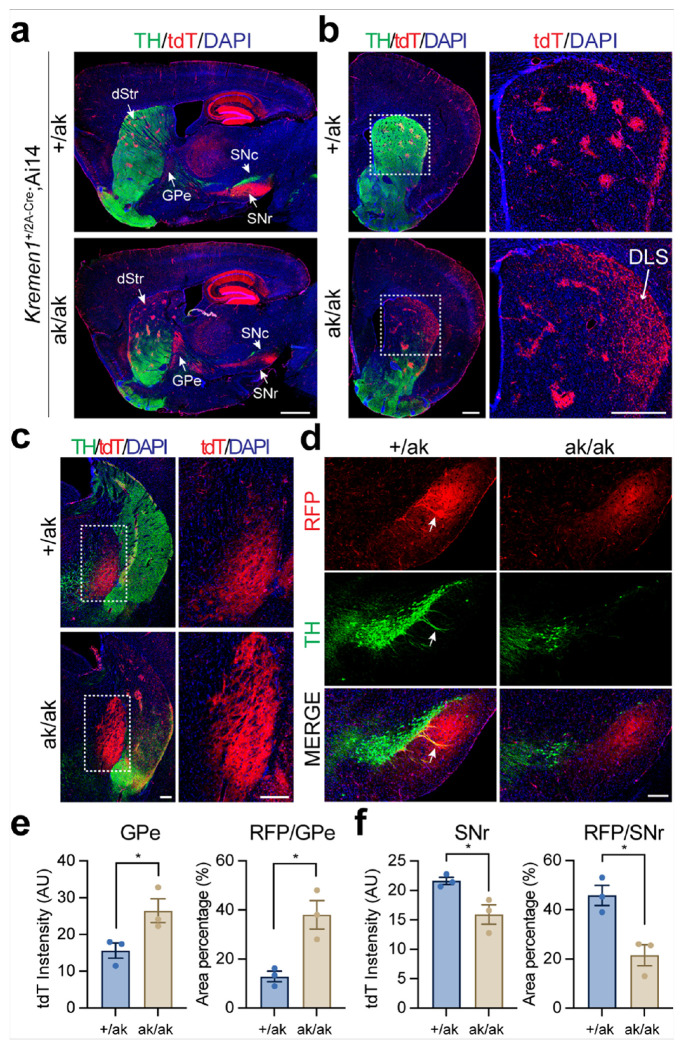
Altered distribution and projection patterns of *Kremen1*^+^ SPNs in the dorsal striatum of *Pitx3*^ak/ak^ mice. **a** Representative sagittal brain sections from *Kremen1*^2A-Cre^; Ai14 mice on *Pitx3*^+/ak^ and *Pitx3*^ak/ak^ backgrounds, stained for TH (green), tdT (red), and DAPI (blue). Scale bar, 1 mm. Abbreviations: dStr, dorsal striatum; GPe, globus pallidus externus; SNc, substantia nigra pars compacta; SNr, substantia nigra pars reticulata; tdT, tdTomato. **b** Coronal sections showing altered tdT signal distribution in the dorsal striatum of *Pitx3*^ak/ak^ mice. Right panel shows a magnified view of the dashed region in the left panel. Arrow points to the dispersed tdT^+^ neurons in DLS. Scale bar, 500 μm. Abbreviations: DLS, dorsolateral striatum. **c** Representative coronal sections showing increased tdT signal in the dorsal GPe of *Pitx3*^ak/ak^ mice. Right panel shows a magnified view of the dashed area. Scale bar, 200 μm. **d** Reduced tdT signal in the SNr of *Pitx3*^ak/ak^ mice. Arrows indicate dendron-bouquet structures. Scale bar, 200 μm. **e** Quantification of average tdT signal intensity and the percentage of tdT-positive area in the GPe. Unpaired t test, n = 3 mice per genotype. Signal intensity: t(4) = 2.818, *p = 0.0479; positive area: t(4) = 4.082, *p = 0.0151. **f** Quantification of average tdT signal intensity and the percentage of tdT-positive area in the SNr. Unpaired t test, n = 3 mice per genotype. Signal intensity: t(4) = 3.231, *p =0.0319; positive area: t(4) = 4.129, *p = 0.0145. Data are presented as mean ± SEM. *p < 0.05 indicates statistical significance.

To assess the output pathways of patchy SPNs, we generated *Kremen1*^2A – Cre^; Ai14 bigenic mice on *Pitx3*^+/ak^ or *Pitx3*^ak/ak^ backgrounds, enabling selective labeling of *Kremen1*^+^ patchy SPNs with tdT. In *Pitx3*^ak/ak^ mice, axonal projections to the GPe were increased, whereas projections to the SNr were markedly reduced compared to controls (Fig. 4a). Moreover, patchy SPNs in these mice appeared more dispersed and lacked the characteristic clustered organization within the superficial dorsolateral striatum (DLS) (arrow, Fig. 4b). The increase in projection was localized primarily to the dorsal GPe (Fig. 4c); while dendron-bouquet structures, formed by the dendrites of DANs and axons of patchy dSPN [43], were entirely absent in the SNr (Fig. 4d). Quantitative analyses of serial GPe and SNr sections confirmed these projection changes (Fig, 4e, 4f).

**Figure 5:**
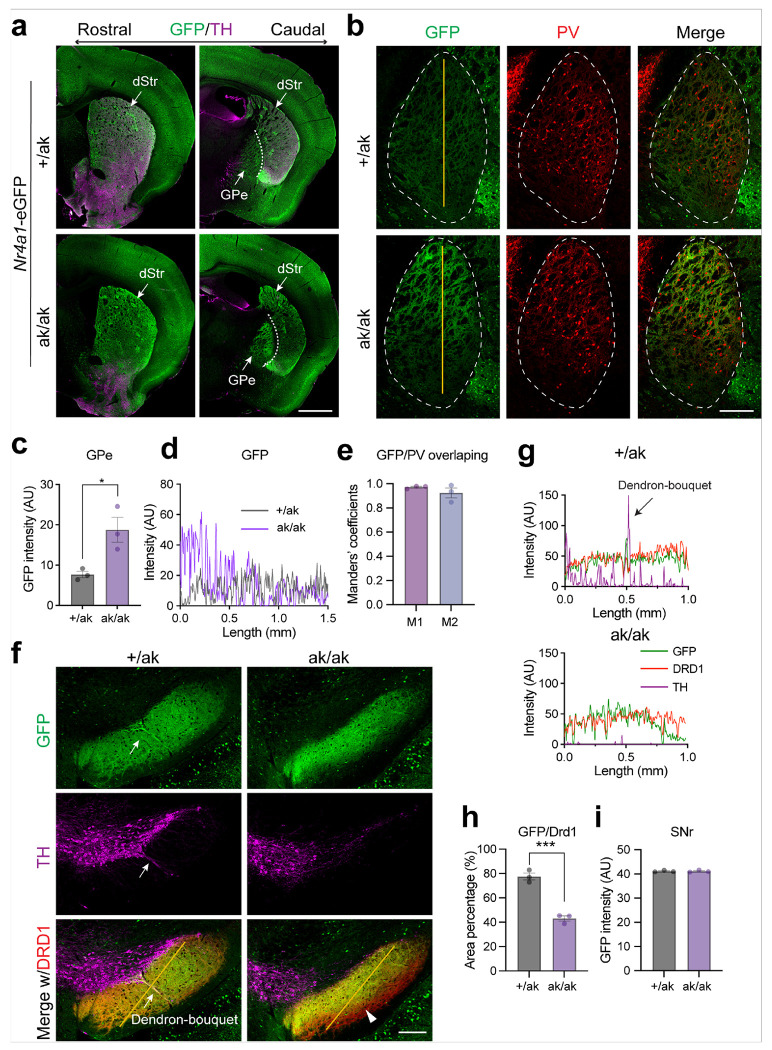
Altered distribution and projection patterns of patchy SPNs in *Nr4a1*-eGFP mice on a *Pix3*^ak/ak^ background. **a** Representative coronal sections from rostral to caudal levels of *Nr4a1*-eGFP mice on *Pitx3*^+/ak^ and *Pitx3*^ak/ak^ backgrounds, stained for TH (magenta). Note the altered GFP signal distribution in the dorsal striatum and dorsal GPe of *Pitx3*^ak/ak^ mice. Scale bar, 1 mm. Abbreviations: dStr, dorsal striatum; GPe, globus pallidus externus. **b** GFP signal distribution in the GPe of *Pitx3*^ak/ak^ mice merged with PV (red) expression in the GPe. Dotted lines outline the GPe. Solid lines indicate the regions used for intensity analysis in **d**. Scale bar, 250 μm. **c** Quantification of average GFP signal intensity in the GPe of *Pitx3*^+/ak^ and *Pitx3*^ak/ak^ mice (n = 3 mice per genotype). Unpaired t test, t(4) = 3.465, *p = 0.0257. **d** Intensity profile analysis of GFP signals along the solid lines in **b**. GFP signal intensity is higher in the dorsal GPe of *Pitx3*^ak/ak^ mice (magenta) compared to the controls (gray). **e** Colocalization analysis of GFP and PV expression in the GPe of *Pitx3*^ak/ak^ mice (n = 3 mice). M1 = 0.97 ± 0.0007; M2 = 0.92 ± 0.04. M1: the proportion of PV signal overlapping with GFP. M2: the proportion of GFP signal overlapping with PV. **f** Representative coronal SNr sections from *Nr4a1*-eGFP mice on *Pitx3*^+/ak^ and *Pitx3*^ak/ak^ backgrounds, stained for TH (magenta) and DRD1 (red). DRD1 was used to outline the SNr. Arrows indicate dendron-bouquet structure in *Pitx3*^+/ak^ mice; arrowhead indicates reduced GFP signal in the ventral SNr. Solid lines indicate the regions used for intensity analysis in **g.** **g** Intensity profile analysis of GFP signals along the solid lines in the SNr from *Pitx3*^+/ak^ (top panel) and *Pitx3*^ak/ak^ (bottom panel) mice in **f**. **h** Percentage of GFP-expressing area within the SNr (Drd1^+^ area) in *Pitx3*^+/ak^ and *Pitx3*^ak/ak^ mice (n = 3 mice per each genotype). Unpaired t test, t(4) = 9.578, ***p = 0.0007. **g** Quantification of average GFP signal intensity in the SNr of *Pitx3*^+/ak^ and *Pitx3*^ak/ak^ mice (n = 3 mice per genotype). Unpaired t test, t(4) = 0.07197, p = 0.9461. Data are presented as mean ± SEM. ***p < 0.001; *p < 0.05.

**Figure 6:**
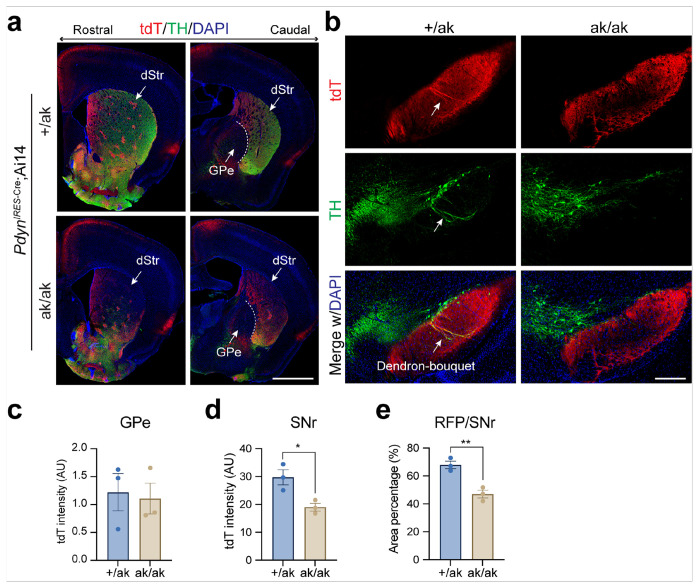
Altered distribution and projection patterns of patchy SPNs in the dorsal striatum of *Pdyn*^IRES-Cre^; Ai14 mice on a *Pix3*^ak/ak^ background. **a** Representative coronal sections from rostral to caudal levels of *Pdyn* ^IRES-Cre^; Ai14 mice on *Pitx3*^+/ak^ and *Pitx3*^ak/ak^ backgrounds, stained for TH (green) and DAPI (blue). Scale bar, 1 mm. Abbreviations: dStr, dorsal striatum; GPe, globus pallidus externus. **b** Representative coronal SNr sections from *Pdyn* ^IRES-Cre^; Ai14 mice on *Pitx3*^+/ak^ and *Pitx3*^ak/ak^ backgrounds, stained for TH (magenta) and DAPI (blue). Arrows indicate dendron-bouquet structures in *Pitx3*^+/ak^ mice. Scale bar, 200 μm. **c** Quantification of average tdT signal intensity in the GPe of *Pitx3*^+/ak^ and *Pitx3*^ak/ak^ mice (n = 3 mice per genotype). Unpaired t test, t(4) = 0.26, p = 0.8077. **d** Quantification of average tdT signal intensity in the SNr of *Pitx3*^+/ak^ and *Pitx3*^ak/ak^ mice (n = 3 mice per genotype). Unpaired t test, t(4) = 3.51, *p = 0.0247. **e** Percentage of tdT-expressing area within the SNr in *Pitx3*^+/ak^ and *Pitx3*^ak/ak^ mice (n = 3 mice per each genotype). Unpaired t test, t(4) = 5.406, **p = 0.0057. Data are presented as mean ± SEM. **P<0.01; *p < 0.05.

Similar alterations in both somatic distribution in the DLS and projection patterns in GPe and SNr were observed in *Nr4a1*-eGFP; *Pitx3*^ak/ak^ mice (Fig. 5a–i). Given that dSPNs can send axonal collaterals to the GPe [44], we next examined whether increased dSPN collateralization might account for the enhanced GPe projections. To test this, we analyzed *Pdyn* ^IRES–Cre^; Ai14 mice, in which tdT preferentially labels patchy dSPNs [45, 46]. In these mice, PITX3 loss did not affect GPe projections but still led to a reduction in SNr projections (Fig. 6a–e). To further investigate the contribution of dSPN collaterals, we examined *Nr4a1*-eGFP; *Drd1*-tdT mice and found no difference in tdT signal intensity in the GPe of *Pitx3*^ak/ak^ and control mice (**Supplementary Fig. S3**).

Taken together, these complementary datasets suggest that PITX3 deficiency disrupts the balance of patchy SPN output pathways by reducing SNr projections due to a loss of patchy dSPNs and increasing GPe projections through an expansion of patchy iSPNs.

### Optogenetic stimulation of patchy SPNs reveals reversed motor effects in Pitx3^ak/ak^ mice

**Figure 7:**
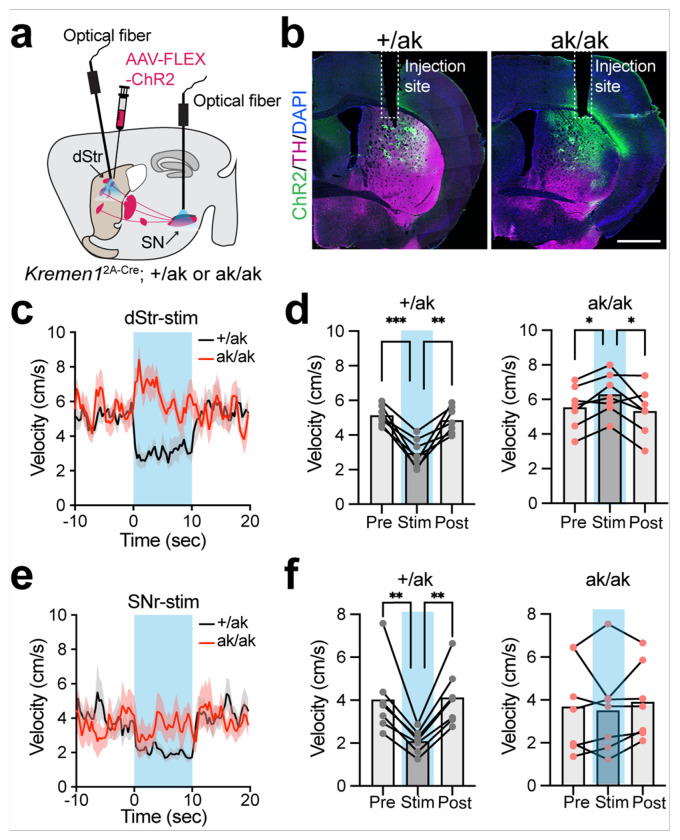
Optogenetic activation of *Kremen1*^+^ SPNs in the dorsal striatum promote locomotion in *Pitx3*^ak/ak^ mice. **a** Schematic illustrating AAV-FLEX-ChR2 vector injection and optical fiber implantation for selective activation of *Kremen1*^+^ SPNs in the dorsal striatum or the SN in *Pitx3*^+/ak^ and *Pitx3*^ak/ak^ mice. **b** Representative coronal images showing ChR2 (green), TH (magenta) and DAPI (blue) expression in the dStr. Fiber implant locations in the dStr are marked. Scale bars: 1 mm. **c** Instantaneous velocity aligned to optogenetics stimulations (10s, blue shaded area) of *Kremen1*^+^ SPNs in the dorsal striatum. **d** Average velocity during pre-stimulation (Pre), stimulation (Stim, blue shaded area), and post-stimulation (Post) in *Pitx3*^+/ak^ mice (left) and *Pitx3*^ak/ak^ mice (right) from panel **c**. For *Pitx3*^+/ak^ mice: Pre = 5.14 ± 0.21 cm/s, Stim = 2.96 ± 0.31 cm/s, Post = 4.86 ± 0.29 cm/s, n=7 mice; one-way ANOVA with multiple comparisons, Pre vs. Stim, ***p = 0.0009, Stim vs. Post, **p = 0.0038. For *Pitx3*^ak/ak^ mice: Pre = 5.54 ± 0.47 cm/s, Stim = 6.29 ± 0.46 cm/s, Post = 5.33 ± 0.53 cm/s, n=7 mice; one-way ANOVA with multiple comparisons, Pre vs. Stim, *p = 0.0169, Stim vs. Post, *p = 0.0365. Error bars represent mean ± SEM. **e** Instantaneous velocity aligned to optogenetics stimulations (10s, blue shaded area) of *Kremen1*^+^ dSPNs in the SN. **f** Average velocity during pre-stimulation (Pre), stimulation (Stim, blue shaded area), and post-stimulation (Post) in *Pitx3*^+/ak^ mice (left) and *Pitx3*^ak/ak^ mice (right) from panel **e**. For *Pitx3*^+/ak^ mice: Pre = 4.04 ± 0.64 cm/s, Stim = 2.09 ± 0.20 cm/s, Post = 4.12 ± 0.51 cm/s, n=7 mice; one-way ANOVA with multiple comparisons, Pre vs. Stim, **p = 0.0065, Stim vs. Post, **p = 0.0017. For *Pitx3*^ak/ak^ mice: Pre = 3.69 ± 0.80 cm/s, Stim = 3.51 ± 0.80 cm/s, Post = 3.92 ± 0.67 cm/s, n=7 mice; one-way ANOVA with multiple comparisons, Pre vs. Stim, p = 0.70, Stim vs. Post, p = 0.44. Error bars represent mean ± SEM.

Recent studies have shown that patchy dSPNs suppress locomotion via inhibition of ALDH1A1^+^ DANs [33, 34], whereas patchy indirect pathway SPNs (iSPNs) exert a weaker locomotion-promoting effect [33, 47]. Under normal conditions, the net outcome of patchy SPN activation is locomotion inhibition [33]. To examine the functional consequences of patchy SPN reorganization in *Pitx3*^ak/ak^ mice, we performed optogenetic stimulation to activate either both patchy dSPNs and iSPNs or dSPNs alone (Fig. 7a, 7b). In control mice, stimulation of both patchy SPN subtypes significantly reduced locomotor velocity (Fig. 7c, 7d), consistent with the dominant inhibitory role of patchy dSPNs [33]. In contrast, the same stimulation paradigm in *Pitx3*^ak/ak^ mice resulted in a marked increase in locomotor speed (Fig. 7c, 7d), indicating a reversal of functional output. Notably, direct stimulation of patchy dSPNs alone failed to suppress locomotion in *Pitx3*^ak/ak^ mice, whereas it reliably reduced movement in controls (Fig. 7e, 7f). These findings suggest that the loss of ALDH1A1^+^ SNc DANs in *Pitx3*^ak/ak^ mice abolishes the suppressive function of patchy dSPNs and instead enhances the locomotion-promoting infiuence of patchy iSPNs, leading to a paradoxical locomotor enhancement upon patchy SPN activation. This functional reversal likely contributes to the hyperactivity phenotype observed in *Pitx3*^ak/ak^ mice.

## Discussion

By integrating molecular mapping, anatomical tracing, and optogenetic manipulation, we reveal a previously unrecognized form of developmental circuit plasticity in the dorsal striatum of *Pitx3*^ak/ak^ mice triggered by the selective loss of ALDH1A1^+^ SNc DANs, a subpopulation preferentially vulnerable in PD [8, 13]. Our findings demonstrate a selective reorganization of patchy SPNs, marked by a pronounced reduction in the ratio of patchy dSPNs to patchy iSPNs and a reversal of their net infiuence on motor output. These results suggest that early dopaminergic depletion drives enduring structural and functional adaptations in basal ganglia circuits, which may contribute to altered motor behaviors.

*Pitx3*^ak/ak^ mice exhibit a profound loss of ALDH1A1^+^ SNc DANs during development [18, 48]; paradoxically, however, they display hyperlocomotion [19, 49], in stark contrast to the bradykinesia observed following ablation or inhibition ALDH1A1^+^ SNc DAN in adult animals [12, 50]. This discrepancy suggests that developmental dopamine loss may engage compensatory mechanisms within the striatum. While prior studies have primarily focused on morphological and electrophysiological alterations in SPNs [49, 51–53], the specific reorganization of patchy SPNs has not been well characterized. Given that patchy dSPNs form reciprocal connections with ALDH1A1^+^ SNc DANs, the developmental loss of this DAN subtype likely leads to a reduction in patchy dSPNs due to diminished trophic support. Consistent with this notion, we observed a marked decrease in the number of patchy dSPNs in the dorsal striatum and a corresponding loss of their projections to the SNr. Unexpectedly, we also detected a selective increase in patchy iSPNs, resulting in a reversal of the patchy dSPN:iSPN ratio from greater than 1 in controls to less than 1 in *Pitx3*^ak/ak^ mutants. This shift likely refiects dopamine-dependent regulation of SPN subtype differentiation, survival, or connectivity during early striatal circuit assembly [51, 54, 55]. The molecular mechanisms underlying the altered SPN composition remain to be elucidated. Whether these changes arise from fate specification or differential survival is unclear. Single-cell transcriptomic profiling of SPNs in *Pitx3*^ak/ak^ mice may help define subtype-specific gene expression changes in response to developmental dopamine loss.

**Figure 8:**
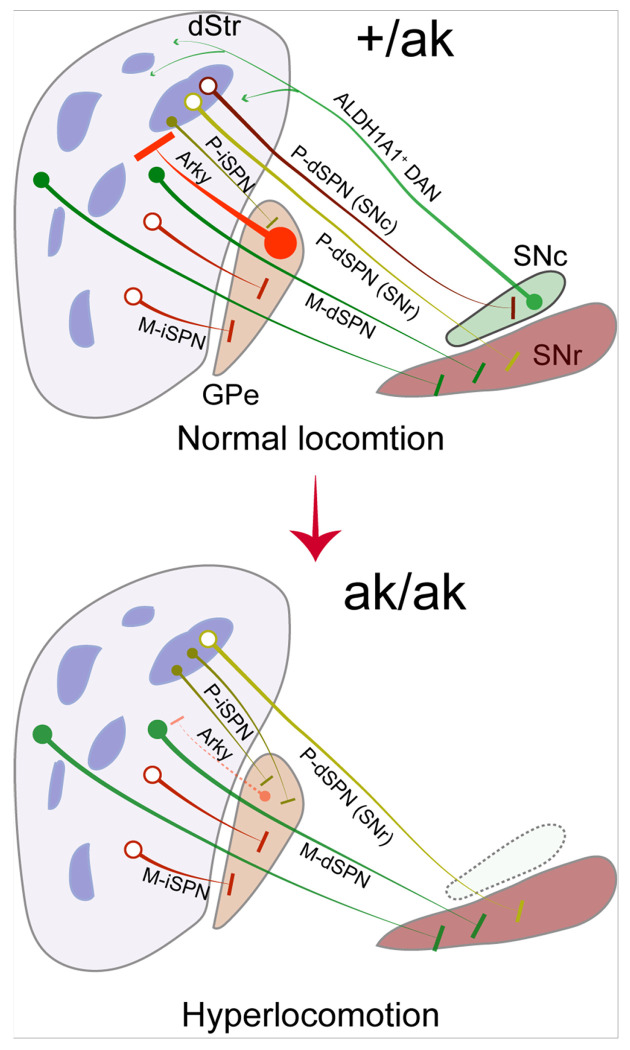
Model of patchy SPN reorganization in *Pitx3*^ak/ak^ mice. This schematic illustrates a selective reorganization of patchy SPNs in *Pitx3*^ak/ak^ mice, characterized by a marked reduction in the ratio of patchy dSPNs to patchy iSPNs, and a reversal of their net effect on motor output due to early dopaminergic depletion. The loss of SNc-projecting patchy dSPNs may abolish their locomotor-suppressing effect, whereas the increased projections of patchy iSPNs to the GPe may enhance inhibition of Arky neurons, thereby weakening their inhibitory feedback to striatal SPNs, including matrix dSPNs. This resulting disinhibition of matrix dSPNs likely contribute to the hyperlocomotion observed in *Pitx3*^ak/ak^ mice, particularly under conditions of heightened patchy iSPN activity.

Anatomical tracing in multiple reporter lines confirmed that the shift in patchy SPN subtype ratio is accompanied by altered projection patterns. In *Kremen1*^2A – Cre^; Ai14 mice, *Pitx3*^ak/ak^ mutants exhibited enhanced projections from patchy SPNs to the GPe and reduced projections to the SNr. These findings were replicated in the *Nr4a1*-eGFP reporter line, supporting the conclusion that the patchy iSPN population is selectively expanded and disproportionately contributes to GPe innervation. Further analyses using *Pdyn*^IRES–Cre^; Ai14 and *Nr4a1*-eGFP; *Drd1*-tdT mice confirmed that patchy dSPNs selectively lost their projections to SNr, with no significant collateralization to GPe. These projection alterations support a model (Fig. 8), in which early developmental dopamine loss leads to a reorganization of SPN connectivity that favors indirect pathway output from patch compartments.

Optogenetic stimulation provided strong functional validation of the anatomical findings in *Pitx3*^ak/ak^ mice. In control mice, activation of patchy SPNs, especially dSPNs, suppressed locomotor activity, consistent with their net inhibitory role on ALDH1A1^+^ DANs and downstream motor circuits [33, 34, 47]. However, in *Pitx3*^ak/ak^ mice, the same stimulation paradigm increased locomotor speed, suggesting a functional reversal of patchy SPN output. Moreover, selective activation of patchy dSPNs alone failed to suppress movement in *Pitx3*^ak/ak^ mice, reinforcing the idea that these cells are, reduced in number, or have lost effective downstream connectivity. Meanwhile, the enhanced locomotion observed during activation of patchy iSPNs aligns with their increased proportion and GPe innervation. Together, these findings indicate that the hyperactivity observed in *Pitx3*^ak/ak^ mice likely stems from an imbalance in patchy SPN circuitry, skewed toward indirect pathway dominance.

Most prior studies have attributed the locomotor effects of patchy SPNs to their modulation of DAN activity, particularly ALDH1A1^+^ SNc DANs [33, 34, 47]. In the absence of these DANs, as in *Pitx3*^ak/ak^ mice, how might reorganized patchy SPNs regulate locomotion? An early study using an independent reporter line demonstrated that patchy iSPNs preferentially target a central subregion of the GPe [47], which corresponds to a distinct subpopulation of GPe neurons associated with arkypallidal (Arky) cells, commonly marked by Npas1 or FoxP2 [56–58]. Unlike prototypical GPe neurons that engage the indirect pathway via the subthalamus nuclei, Arky neurons project back to the striatum, where they provide potent inhibition to SPNs [59–61]. The observed increase in patchy iSPN projections to the GPe in *Pitx3*^ak/ak^ mice may result in enhanced inhibition of Arky neurons, thereby reducing their inhibitory feedback to striatal SPNs, including matrix dSPNs (Fig. 8). This disinhibition of matrix dSPNs may contribute to the hyperlocomotion observed in *Pitx3*^ak/ak^ mice, particularly under conditions of increased patchy iSPN activity. Future studies employing electrophysiological recordings and circuit-mapping approaches will be essential to delineate how patchy iSPNs modulate Arky neuron activity in the GPe of *Pitx3*^ak/ak^ mice.

Although PD is defined by progressive dopaminergic degeneration, compensatory remodeling of downstream circuits may occur, particularly in early or preclinical stages. The observed increase in patchy iSPN activity may reflect an adaptive mechanism aimed at sustaining motor output in the context of dopamine loss. Despite these important insights, several limitations should be noted. First, the loss of ALDH1A1^+^ SNc DAN in *Pitx3*^ak/ak^ mice occurs during early postnatal development. Thus, the circuit adaptations observed in this model likely reflect developmental plasticity, which may differ fundamentally from the compensatory changes that emerge in adult-onset models or in human PD. Whether similar circuit remodeling occurs in progressive PD models or in patients remains to be determined. Second, although *Pitx3*^ak/ak^ mice display hyperactivity in open-field tests, they exhibit deficits in motor skill learning [53, 62], suggesting that compensatory changes in the striatum are insufficient to fully restore complex motor functions. Third, our analysis was limited to anatomical projections, optogenetics manipulation and behavioral output. Future studies incorporating electrophysiological and neurochemical approaches will be critical to elucidate the functional consequences of these circuit alterations in the dorsal striatum and GPe.

## Conclusion

We identify a novel form of striatal circuit remodeling following the developmental loss of ALDH1A1^+^ SNc DANs in mice. This reorganization selectively alters the balance of patchy SPNs, resulting in a shift toward indirect pathway dominance and functional reversal in their motor output. These findings provide mechanistic insight into how early dopamine depletion can reshape basal ganglia circuitry and highlight patchy iSPNs as potential targets for therapeutic intervention in PD.

## Supplementary Material

This is a list of supplementary files associated with this preprint. Click to download.

• Supp20250818.docx

## Data Availability

All data generated or analyzed during this study are included in this published article. Source data provided with this paper.

## Abbreviations

PD: Parkinson’s disease
DANs: Dopaminergic neurons
SNc: Substantia nigra pars compacta
VTA: Ventral tegmental area
ALDH1A1: Aldehyde dehydrogenase 1 family member A1
SPNs: Striatal projection neurons
dSPNs: Direct-pathway striatal projection neurons
iSPNs: Indirect-pathway striatal projection neurons
GPe: Globus pallidus externa
GPi: Globus pallidus interna
SNr: Substantia nigra pars reticulata
AAV: Adeno-associated virus
ChR2: Channelrhodopsin-2
tdT: tdTomato fluorescent protein
TH: Tyrosine hydroxylase
CTIP2: COUP-TF-interacting protein 2
EYFP: Enhanced yellow fluorescent protein
Drd1: Dopamine receptor D1
Drd2: Dopamine receptor D2
